# Role of MicroRNA-26b in Glioma Development and Its Mediated Regulation on EphA2

**DOI:** 10.1371/journal.pone.0016264

**Published:** 2011-01-14

**Authors:** Ning Wu, Xiangzhong Zhao, Ming Liu, Haizhou Liu, Weicheng Yao, Yuyan Zhang, Shousong Cao, Xiukun Lin

**Affiliations:** 1 Institute of Oceanology, Chinese Academy of Sciences, Qingdao, China; 2 Brain Institute, Affiliated Hospital of Qingdao Medical University, Qingdao, China; 3 Roswell Park Cancer Institute, Buffalo, New York, United States of America; 4 Graduate School, Chinese Academy of Sciences, Beijing, China; Lehigh University, United States of America

## Abstract

**Background:**

MicroRNAs (miRNAs) are short, non-coding RNAs that regulate the expression of multiple target genes. Deregulation of miRNAs is common in human tumorigenesis. Low level expression of miR-26b has been found in glioma cells. However, its underlying mechanism of action has not been determined.

**Methodology/Principal Findings:**

Real-time PCR was employed to measure the expression level of miR-26b in glioma patients and cells. The level of miR-26b was inversely correlated with the grade of glioma. Ectopic expression of miR-26b inhibited the proliferation, migration and invasion of human glioma cells. A binding site for miR-26b was identified in the 3′UTR of *EphA2*. Over-expression of miR-26b in glioma cells repressed the endogenous level of *EphA2* protein. Vasculogenic mimicry (VM) experiments were performed to further confirm the effects of miR-26b on the regulation of *EphA2*, and the results showed that miR-26b inhibited the VM processes which regulated by *EphA2*.

**Significance:**

This study demonstrated that miR-26b may act as a tumor suppressor in glioma and it directly regulates *EphA2* expression. *EphA2* is a direct target of miR-26b, and the down-regulation of *EphA2* mediated by miR-26b is dependent on the binding of miR-26b to a specific response element of microRNA in the 3′UTR region of *EphA2* mRNA.

## Introduction

MicroRNAs (miRNAs) are short single stranded RNA molecules, which serve as master regulators of gene expression. miRNAs regulate gene expression in a sequence-specific fashion; miRNAs bind to 3′untranslated regions (UTRs) of mRNAs and then affect the translation and/or stability of that mRNA, leading to a reduction in protein levels. Tumors analyzed by miRNA profiling have exhibited significantly distinct miRNA signatures compared to normal cells from the same tissue [1], [2]. The abnormal levels of miRNAs in tumors have important pathogenetic consequences [3]. Some miRNAs are over-expressed in tumors and act as oncogenes, promoting tumor aggravation by down-regulating tumor suppressors [4]. For example, the miR-17-miR-92 cluster in T-cell acute lymphoblastic leukemia reduces the level of the transcription factor *E2F1* [5], [6]; miR-21 down-regulates the tumor-inhibiting factor *PTEN* in lung cancer cells; and miR-125b is an important repressor of *p53* and inhibits p53-induced apoptosis in human neuroblastoma cells [7]. On the other hand, tumors lost miRNAs generally participate in oncogene over-expression. For example, the let-7 family represses *Ras* and *Myc* oncogenes in cancers [8], [9], and the miR-15-miR-16-1 cluster down-regulates *Bcl-2* and induces apoptosis in a leukemic cell line model [10].

miR-26b is one of the miRNAs involving in the response to hypoxia, a well documented tumor microenvironment factor [11]. Recent study confirmed that the expression of miR-26b was changed in several human cancer cell lines including glioma cells, [12]. miRNA profile analyses revealed that miR-26b was one of the significantly decreased miRNAs in glioma cells compared to normal brain tissues [12]. However, the role of miR-26b in glioma development has not been well documented and little is known about its target genes. Additionally, the effect of abnormal expression of miR-26b on tumor grade needs to be addressed.

Erythropoietin-producing hepatocellular (EPH) receptors and their Ephrin ligands constitute the largest sub-family of receptor tyrosine kinases (RTKs), which are involved in many biological processes and play important roles in disease and development [13]. To date, 14 Eph receptors have been found in mammals. They were divided into two distinct classes, A and B, based on the sequence homology of their extracellular domains. More recently, *EphA* receptors and their corresponding ligands have been implicated in numerous malignancies [14]. Among them, *EphA2* and *ephrinA1* are the most widely studied with respect to development, tumorigenesis, angiogenesis, and metastasis and they may represent as the potential therapeutic targets because of their diverse functions in several types of cancer. Studies have shown that activation of the *EphA2* receptor tyrosine kinase by *ephrinA1* ligands played important roles in cellular signal transduction. [15]. *EphA2* is implicated and functionally altered in a number of cancers and has potential roles in the regulation of cancer cell growth, survival, migration, invasion, and angiogenesis [16], [17], [18]. Increased expression of *EphA2* has been demonstrated in most cancers of epithelial origin, like breast [19], ovarian [17], [20], prostate [21], melanoma [22], esophageal [23], [24], [25], lung carcinomas [26] and brain [27], [28]. Immunohistochemical analysis has revealed that *EphA2* was strongly over-expressed in 90% of GBM patient tumors [27], 85% of prostate adenocarcinomas and 76% of ovarian cancers [18]. Furthermore, the frequent over-expression of *EphA2* in human cancers correlates with poor prognosis and increases metastatic potential [18]. In epithelial cells, ectopic expression of *EphA2* has been shown to result in a malignant phenotype in both in vitro and in vivo experiments [19]. *EphA2* has been proposed as an attractive target for developing novel anticancer therapeutic agents.

In this study, the expression of miR-26b in glioma cells and the tissues from glioma patients with certain grades was studied by real-time PCR analysis. Proliferation, migration, and invasion were analyzed to confirm the effects of miR-26b in glioma cells. The regulation of miR-26b on *EphA2* was confirmed by the experiments of luciferase analysis, Western blotting, and Vasculogenic mimicry (VM) network formation. We found that ectopic expression of miR-26b in U251 and C6 glioma cells resulted in diminished proliferation, migration and invasion activity, accompanied by a low level expression of *EhpA2*. VM formation was also abolished in glioma cells transfected with the miR-26b duplex. Our study provides evidence that miR-26b acts as an anti-oncogene in glioma cells and is an important negative regulator of the *EphA2* gene.

## Results

**Figure 1:**
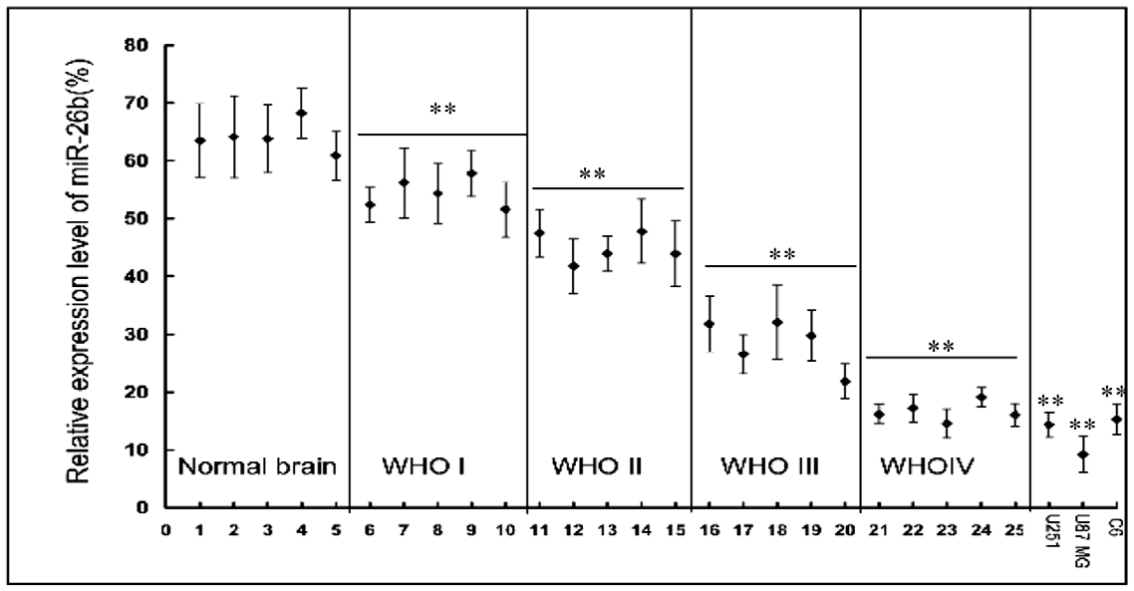
The expression of miR-26b in glioma samples and U251, U87 MG and C6 cells. The grade of glioma was evaluated according to WHO criteria as described in Materials and Methods. Samples ID 1–5 are from normal brain tissues; ID 6–10 from pilocytic astrocytomas classified to WHO I: ID 11–15 from astrocytoma classified to WHO II; ID 16–20 from anaplastic astrocytomas classified to WHO III; and ID 21–25 from Glioblastoma Multiforme classified to WHO IV. Each sample was divided by a dashed line. Total RNA was isolated from the glioma tissues and glioma cells of U251, U87 MG and C6 and real-time PCR was performed to analyze the expression of miR-26b as described in Materials and Methods. The relative expression of miR-26b was expressed as the ratio of the expression level of U6. **P<0.01, as compared to Normal brain tissues group.

The expression of miR-26b in tissues of glioma patients has not been well documented. In order to determine the relationship between miR-26b expression and glioma grades, the expression of miR-26b in normal brain tissues, glioma tumors and glioma cell lines was analyzed by real-time stem-loop RT-PCR. The results showed that in normal brain tissues, miR-26b exhibited a relative high level expression, whereas the expression of miR-26b was significantly (p<0.01) down-regulated in glioma samples (WHO I, WHO II, WHO III and WHO IV). The expression of miR-26b becomes lower with increasing grades of glioma. The expression of miR-26b was also down-regulated in the three tested glioma cell lines, U251, U87 MG and C6 (Fig. 1).

**Figure 2:**
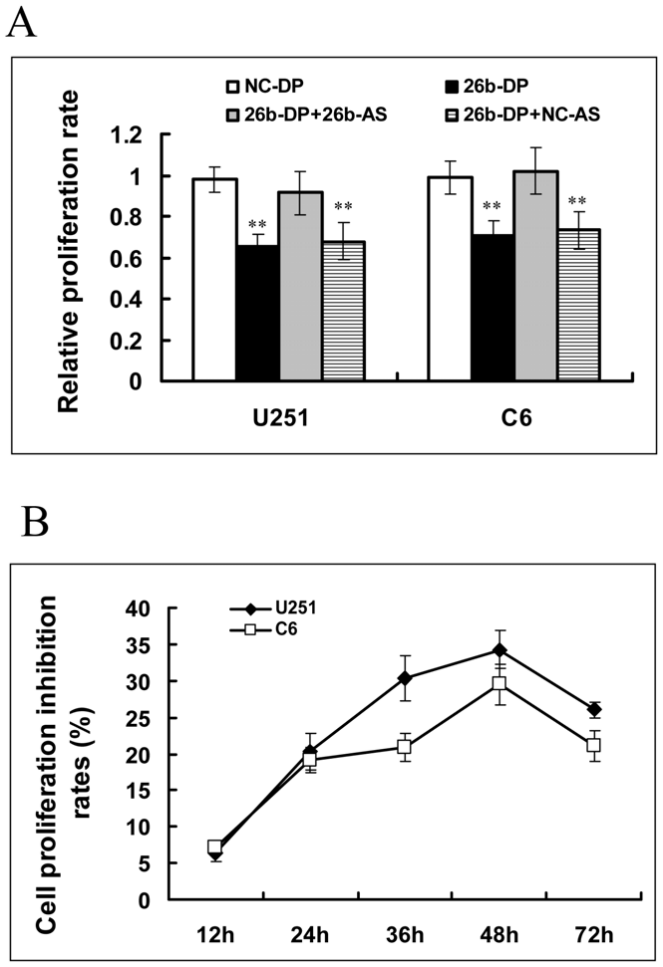
Ectopic expression of miR-26b inhibits glioma cell proliferation in vitro. (A) The proliferation of glioma cell lines, U251 and C6. Cells were first transfected with miR-26b duplex, negative control RNA duplex, co-transfected with 26b-DP and miR-26b specific antisense oligonucleotides (26b-AS) or negative antisense oligonucleotides (NC-AS). After incubation for 48 h, cell proliferation rates were analyzed by MTT assay. The growth rates of cells transfected with NC-DP were defined as 1.0. **P<0.01, as compared to NC-DP group. (B) The cell proliferation inhibition rates of glioma cell lines of U251 and C6 after miR-26b transfection at certain time points. 26b-DP was transfected into U251 or C6 cells, and the cell proliferation inhibition rates were evaluated by MTT assay at 12, 24, 36, 48 and 72 h. The inhibitory rates of cells were the percentage of the ratio of cells proliferation transfected with miR-26b to that transfected with NC-DP.

We next determined the effect of miR-26b on the proliferation of glioma cells. The growth ability of glioma cells was determined by MTT assay. As shown in Fig. 2A, over-expression of miR-26b resulted in the growth inhibition of both U251 human glioma cells and C6 rat glioma cells. However, the growth inhibition induced by miR-26b was abolished when an antisense of miR-26b (26b-AS) was introduced into the cells (Fig. 2A). In contrast, substituting the 26b-AS with a negative control single strand RNA (NC-AS), similar inhibition effect on cell proliferation was found as the transfected 26b-DP alone (Fig. 2A). We examined the inhibitory effect of miR-26b on glioma cells at different time points and found that the maximum inhibition was at the 48 h (Fig. 2B). The inhibition rates were 34.28% and 29.547% in U251 and C6 cells transfected with 26b-DP, respectively (Fig. 2B). These results suggest that miR-26b plays a key role in the proliferation of some glioma cells, and might function as a tumor suppressor in glioma cell lines.

**Figure 3:**
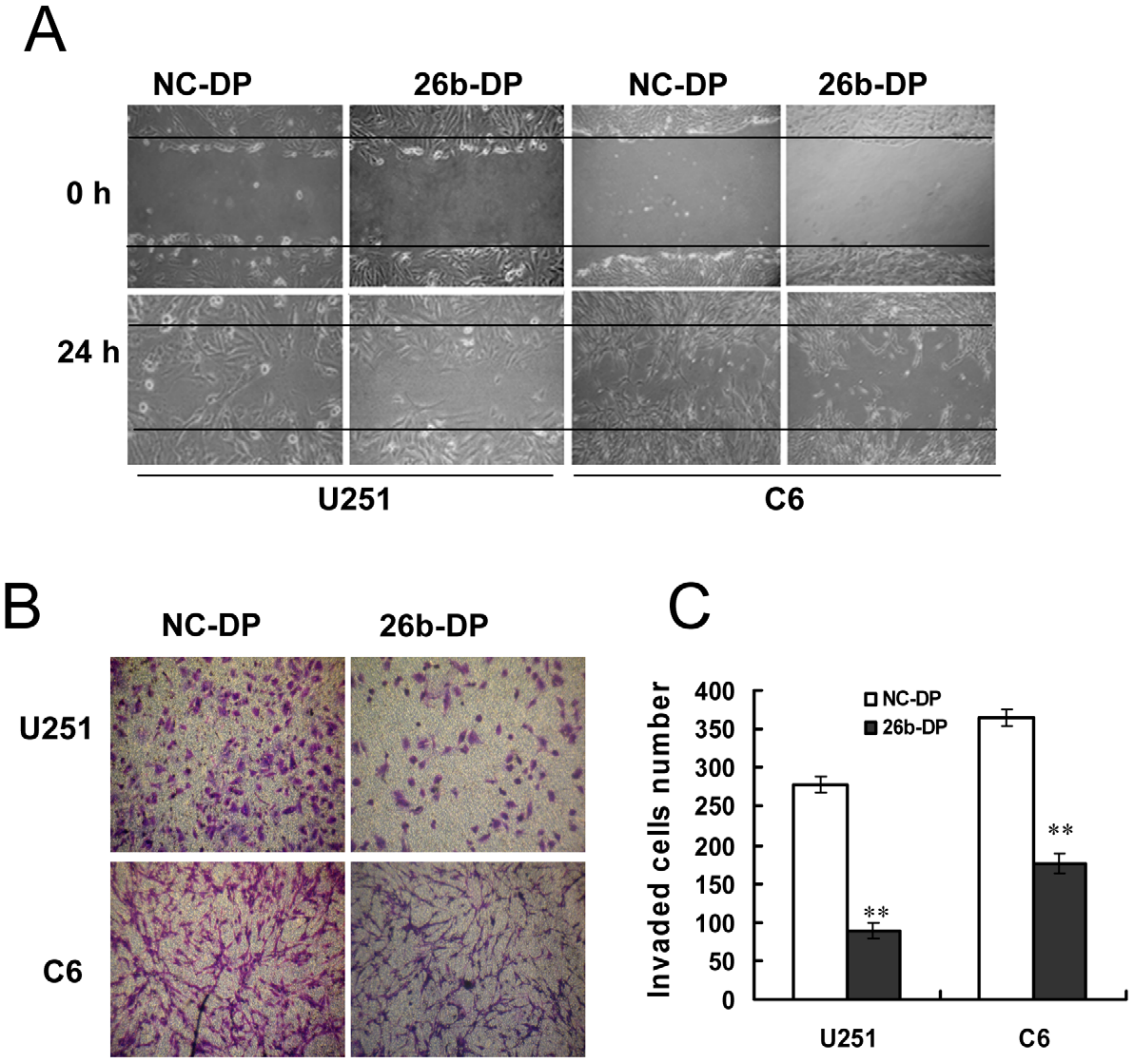
Effect of miR-26b over-expression on glioma cell migration and invasion. (A) Wound healing assay was employed to evaluate the effects of miR-26b on glioma cell migration as described in Materials and Methods. U251 and C6 cells were transfected with 26b-DP or NC-DP at a final concentration of 50 nM/L. When the cell confluence reached about 90% at 48 h post-transfection, an artificial homogenous wound was made as described in Materials and Methods. The boundary of the wound was marked by two solid lines. The experiments were preformed in triplicate and the representative images of photographs at 0 h and 24 h post-wounding are shown at 100× magnification. (B) Effect of miR-26b over-expression on glioma cells invasion. Glioma cells transfected with 26b-DP or NC-DP were plated on Matrigel-coated membranes in the upper chamber of transwells. After incubation for 24 h, non-invading cells on the upper surface of the membrane were removed and the invasive cells on the lower surface were stained with 0.1% Crystal Violet. The stained invasive cells were photographed under an inverted light microscope (100× magnifications). (C) Quantitative results of glioma cell invasion. Invasive cells were quantified by manual counting and the number represents the mean of six counting sights ± SEM. The experiments were performed in triplicate with three independent experiments. Statistically significant differences between the groups of 26b-DP and NC-DP were observed: **P<0.01.

To evaluate the role of miR-26b in glioma cell migration, a wound healing assay was performed. An artificial wound was made 24 h after transfection with 26b-DP, and the cells migrating into the wound were measured after culturing for another 24 h. As shown in Fig. 3A, the migration was significantly decreased in U251 and C6 cells transfected with 26-DP (Fig. 3A). To determine the invasion ability of glioma cells, an in vitro Matrigel invasion assay was employed. As shown in Fig. 3B, cell invasion was markedly reduced in the cells transfected with 26b-DP, exhibiting a 67.98% and 51.78% in reduction of invasion with a statistically significant difference (p<0.01) in the U251 and C6 cells, respectively (Fig. 3C). The results suggest that over-expression of miR-26b inhibits the ability of invasion and migration of glioma cells.

**Figure 4:**
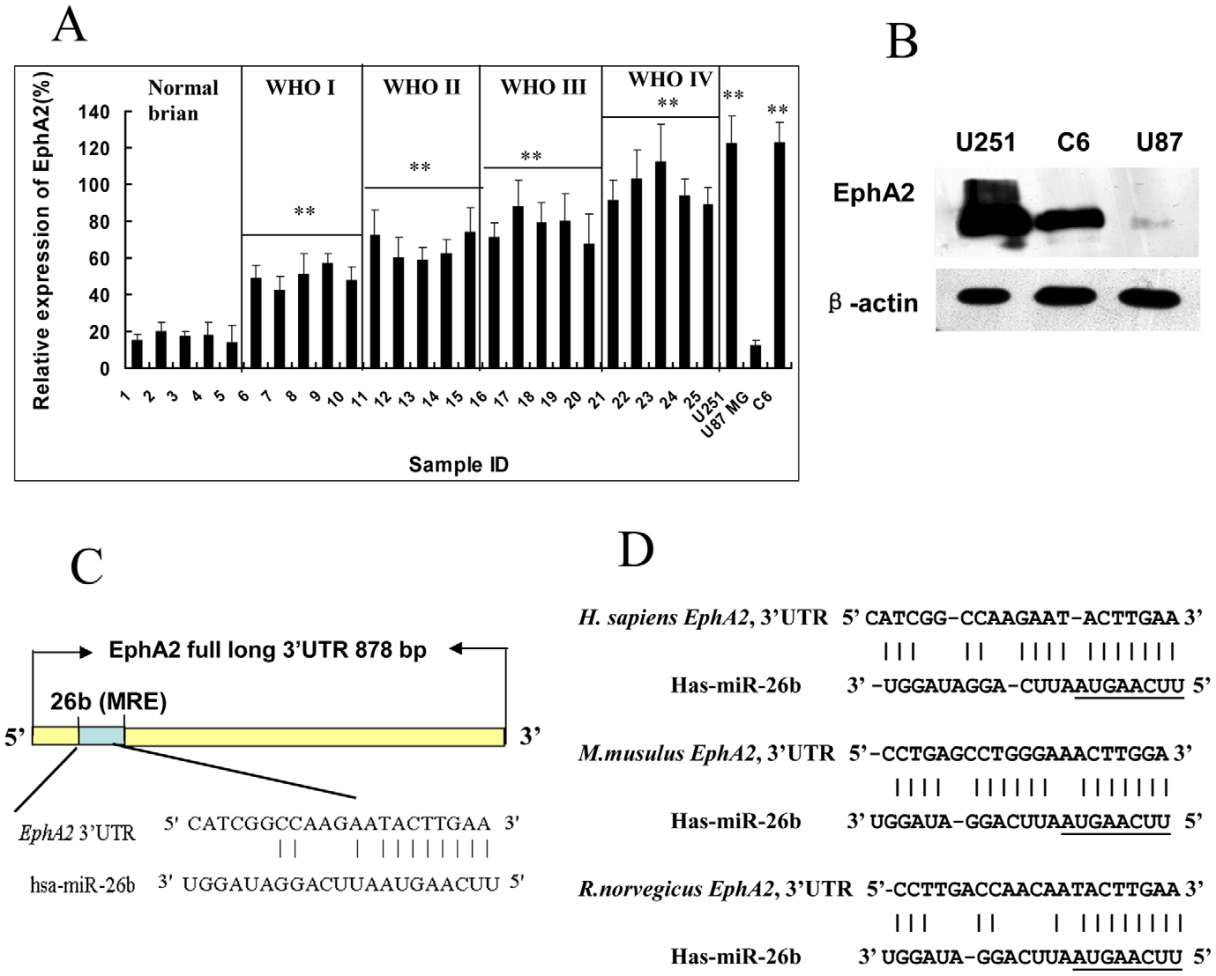
EphA2 is a predicted target of miR-26b. (A) The mRNA expression level of *EphA2* in glioma samples and glioma cell lines. The mRNA expression level of *EphA2* was detected by real-time PCR. The grades of glioma were classified in Materials and Methods. The experiments were preformed more then three times. The relative expression of *EphA2* was the ratio of the expression level of to that of *β-actin*. **P<0.01, as compared to Normal brain tissues group. (B) The protein expression level of *EphA2* in U251, U87 MG and C6 cells. Western blotting analysis was performed to evaluate the expression level of *EphA2* in different glioma cells. *β-actin* was used as a loading control. (C) The shaded region represents the MRE sequences of miR-26b in the 3′UTR of human *EphA2* mRNA as predicted by TargetScan 5.1, miRBase and Pictar. (D) The predicted miR-26b binding site in *EphA2* 3′UTR is highly conserved in mammals. The miR-26b seed sequences and their predicted binding sites in the *EphA2* 3′UTR are shown underlined.

We then tried to find the target genes of miR-26b using the online miRNA target prediction programs miRanda (http://www.microrna.org), TargetScan (http://www. targetscan.org) and PicTar (http://www.pictar.bio.nyu.edu). Approximately 100 targets of miR-26b were predicted from these programs. *EphA2* was of particular interest because previous studies revealed that its expression level was enhanced in approximately 90% of GBM specimens [27], [28]. Therefore, we assumed that the decreased expression of miR-26b resulted in the high level of *EphA2*. Here we detected the expression of *EphA2* in normal brain, glioma samples of different grades, and three glioma cell lines. Indeed, we found that expression of *EphA2* was significantly enhanced with the advance of glioma grade(p<0.01), accompanying the decrease of miR-26b (Fig. 4A). Moreover, the results predicted by TargetScan 5.1, miRBase Target and PicTar showed that there was a specific target site of miR-26b on 3′UTR of *EphA2* (Fig. 4C) and miR-26b was one of the conserved miRNAs targeting *EphA2* in both human and murine (Fig. 4D). The putative miRNA response elements (MREs) of miR-26b were also found in the 3′UTRs of *EphA2* in other vertebrates when compared across distant species (data not shown). These results suggest that miR-26b is likely to be an important regulator of *EphA2*.

**Figure 5:**
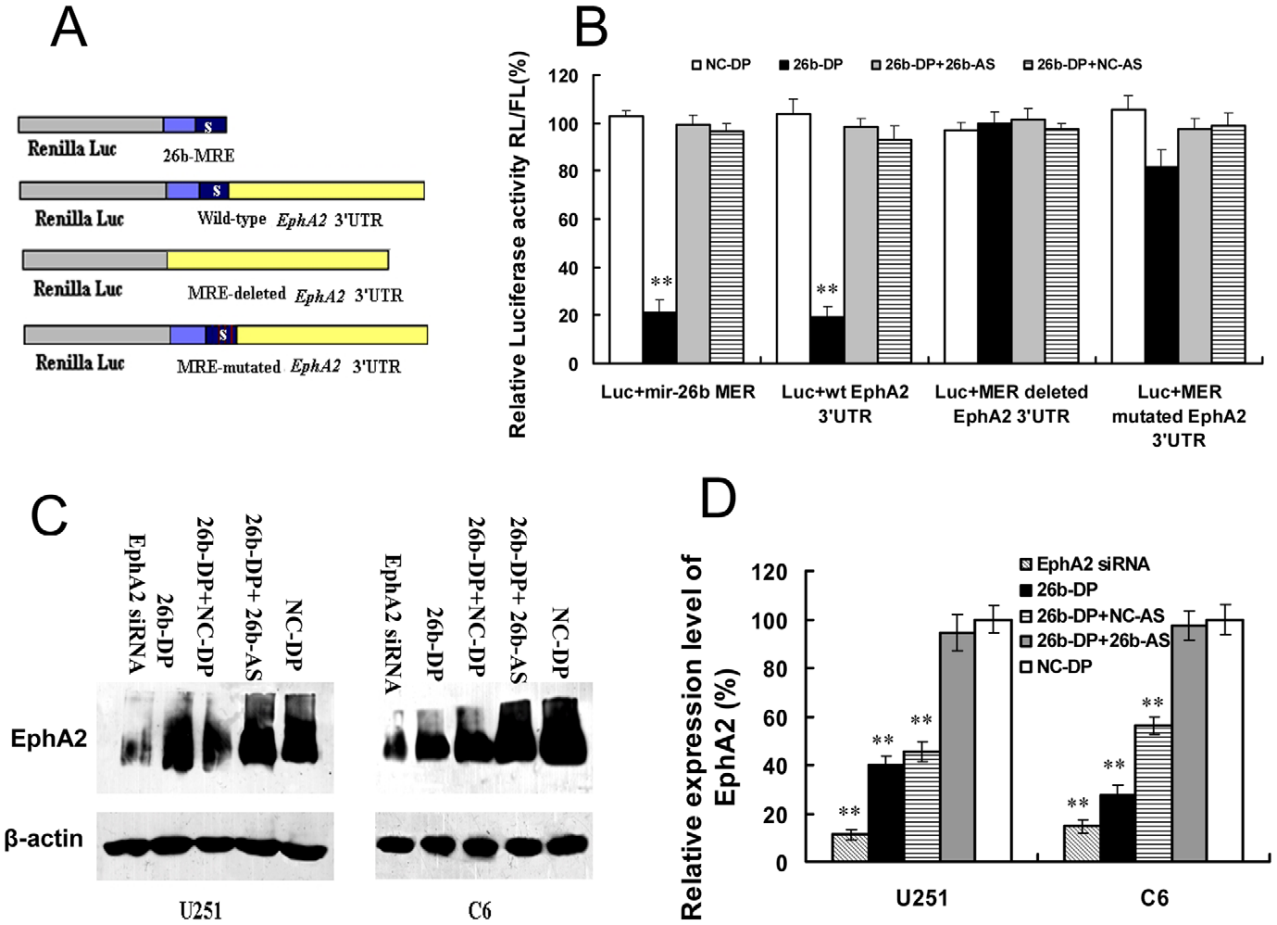
miR-26b binds to the 3′UTR of *EphA2* mRNAs. (A) Synthesis of luciferase reporter assay constructs for validating the interaction of miR-26b with the 3′UTR of *EphA2*. “S” in the Shaded region indicates the “seed” regions in the 26b-MRE of the 3′UTR of *EphA2* mRNAs. The predicted MRE or the whole *EphA2* 3′UTR was inserted into a psiCheck2 vector immediately downstream of the Renilla luciferase gene to construct the luciferase reporter vectors, Luc+miR-26b MRE and Luc+wt *EphA2* 3′UTR, respectively. Luc+MRE deleted 3′UTR was also constructed with a similar approach. A mutation in the seed region of miR-26b MRE of *EphA2* 3′ UTR was made using the Quick change sit-Directed mutagenesis kit to construct the Luc+MRE mutated 3′UTR vector. (B) Quantitative analysis of luciferase activity. HEK-293T cells were transfected with NC-DP, 26-DP, co-transfected with 26b-DP and 26b-AS or NC-AS. After incubation for 48 h, luciferase activity was analyzed as described in Materials and Methods. The activity of Renilla luciferase was normalized to that of the control firefly luciferase in each experiment. The luciferase activity in cells transfected with control miRNA (NC-DP) was defined as 100%. The values represent the Mean ± SEM (n≥6) from at least three independent experiments. The two-tail t-Test was employed to statistically analyze the results. (**) P<0.01 *vs.* NC-DP transfected controls. (C) Over-expression of miR-26b down-regulates *EphA2* expression in U251 and C6 cells. Various miRNAs were transfected into U251 or C6 cells and the level of *EphA2* was analyzed by Western blotting as described in Materials and Methods. (D) Quantitative results of *EphA2* expression. Quantitation of signal intensities was performed using densitometry on a Hewlett-Packard ScanJet 5370 C. The percentage of *EphA2* expression represents the ratio of *EphA2* to β-actin expression (n≥3). The relative expression level of *EphA2* siRNA treated group was used as a positive control. The two-tail t-test was employed to statistically analyze the results: ** P<0.01.

We confirmed the binding of miR-26b to the 3′UTR of human *EphA2* using a luciferase reporter assay (Fig. 5A). Ectopic expression of miR-26b significantly suppressed the luciferase activity in HEK-293 cells co-transfected with miR-26b duplex (26b-DP) and Luc+miR-26b MRE, which contained the miR-26b response element (MRE) region in the 3′UTR of human *EphA2* (Fig. 5B). Similarly, the activity of luciferase in the cells co-transfected with miR-26b and the construct Luc+wt *EPhA2* 3′UTR, which contains the entire 3′UTR region of human *EphA2*, was suppressed by 60∼70% (P<0.01) (Fig. 5B). In contrast, suppression of luciferase activity was almost abolished when the miR-26b MRE was deleted from the 3′UTR of *EphA2* (Fig. 5B). The inhibition of luciferase activity was also greatly decreased when introducing a 3-base mismatch mutation into the seed region of the MRE in the 3′UTR of *EphA2* (Fig. 5A and B). These data indicate that the predicted MRE is critical for the direct and specific binding of miR-26b to *EphA2* mRNA.

To study the regulation of endogenous *EphA2* by miR-26b, 26b-DP was transfected into U251 and C6 cells, both of which express high levels of *EphA2*. An *EphA2* specific siRNA [29] was transfected as a positive control. When the *EphA2* siRNAs were transfected into U251 or C6 cells, the expression level of endogenous *EphA2* proteins was inhibited nearly 90% (Fig. 5C and D). Ectopic expression of miR-26b in U251 cells reduced the level of *EphA2* protein by ∼60% (p<0.01) (Fig. 5C and D). The expression of *EphA2* protein was reduced ∼70% in C6 cells transfected with miR-26b (Fig. 5C and D). These results indicate that miR-26b is a regulator of *Eph2A* in glioma cells, and that the miRNA can down-regulate the expression of *EphA2* at the protein level.

**Figure 6:**
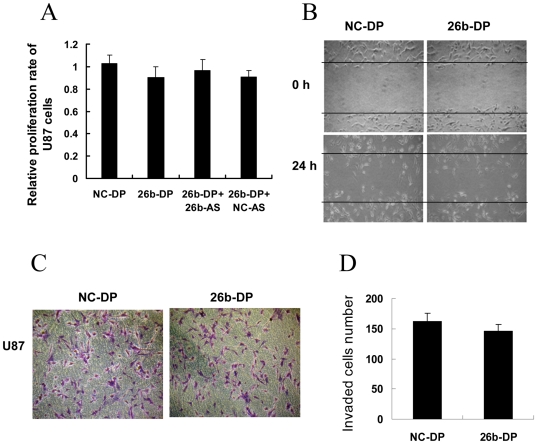
Effects of miR-26b on proliferation, migration and invasion in U87 MG cells. (A) Effect of miR-26b over-expression on U87 MG cell proliferation. U87 MG cells were first transfected with miR-26b duplex, negative control RNA duplex (NC-26DP), co-transfected with 26b-DP and miR-26b specific antisense oligonucleotides (26b-AS), or negative antisense oligonucleotides (NC-AS). Then, the cell proliferation rates were analyzed by MTT assay after 48 h incubation. The growth rates of cells transfected with NC-DP were defined as 1.0. There was no significant difference (P>0.05) among all tested groups. (B) Effect of miR-26b over-expression on migration in U87 MG cells. U87 MG cells were transfected with 26b-DP or NC-DP at a final concentration of 50 nM/L. When the cell confluence reached about 90% at 48 h post-transfection, an artificial homogenous wound was made as described in Materials and Methods. The boundary of the wound was marked by two solid lines. The experiments were preformed in triplicate and the representative images of photographs at 0 h and 24 h post-wounding are shown at magnification of 100×. (C) Effect of miR-26b over-expression on migration and invasion in U87 MG cell. U87 MG cells transfected with 26b-DP or NC-DP were plated on Matrigel-coated membranes in the upper chamber of transwells. After incubation for 24 h, non-invading cells on the upper surface of the membrane were removed and the invasive cells on the lower surface were stained with 0.1% Crystal Violet. The stained invasive cells were photographed under an inverted light microscope (100× magnifications). (D) Quantitative results of glioma cell invasion. Invasive cells were quantified by manual counting and the number represents the mean of six counting sights ± SEM. The experiments were performed in triplicate with three independent experiments.

In order to further confirm the function of miR-26b as an *EphA2* regulator, we next studied the effect of miR-26b in U87 MG glioma cells expressing only low levels of endogenetic *EphA 2* [27] (Fig. 4B). Compared to U251 and C6 which expressed high levels of *EphA2* (Fig. 4A and B), the cell proliferation reduced by miR-26b were not significant (p>0.05) in the U87 MG cells (Fig. 6A). The growth ability of glioma cells in U87 MG, U251, and C6 cells transfected with 26b-DP was reduced with inhibition rates of 10.11% (Fig. 6A), 34.28% and 29.54% (Fig. 2B), respectively, suggesting that the inhibition rates of 26b-DP transfectants were related to endogenous *EphA2* levels in glioma cells. Similarly, the reduction of migration activity in U87 MG cells transfected with 26b-DP was also not notable (Fig. 6B) compared with that in U251 and C6 glioma cells (Fig. 3A). Additionally, as shown in Fig. 3B and 3C, transfection with 26b–DP greatly reduced the ability of U251 and C6 cells to invade Matrigel, exhibiting about 67.98% and 51.78% in reduction of invasion, respectively. However, in U87 MG, only 11.73% reduction was found compared with the cells transfected with scrambled oligonucleotides (NC-DP) (Fig. 6C and D). These results provide solid evidence that miR-26b suppresses glioma cell proliferation, migration and invasion activity in a manner dependent on *EphA2* expression level.

**Figure 7:**
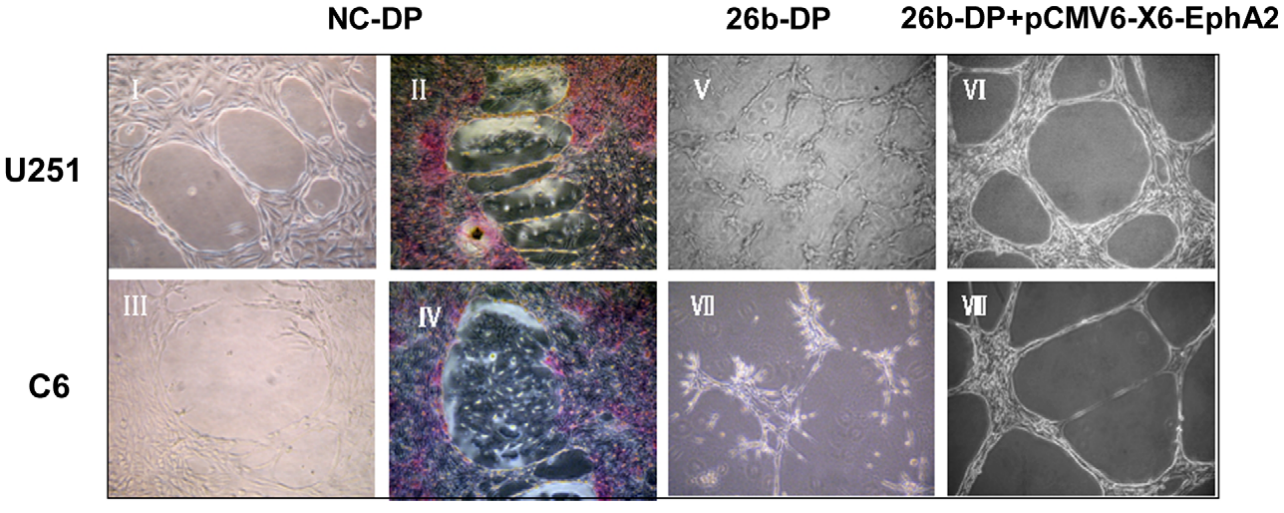
Vasculogenic mimicry (VM) formation assay. U251 cells (I) or C6 cells (III) transfected with negative control microRNA (NC-DP) were seeded onto 6-well plates. ECM Matrigel was dropped onto 18-mm glass coverlips in 6-well tissue culture plate and incubated at 37°C for 30 min and then seeded on the coated coverlips. The VM formation was assessed using an inverted microscope after growth for 24 h. II and IV represent the U251 and C6 network formation stained by PAS, respectively. U251 (V) and C6 (VI) cells were transfected with 26b-DP, the transfected cells were seeded on the coated coverlips after 24 h incubation. The VM formation was assessed using an inverted microscope after growth for another 24 h. The U251 and C6 cells tansfected with 26b-DP first, then *EphA2* expression vector which not include the 3′UTR (pCMV6-AC-GFP-EphA2) was co-transfected after 24 h incubation, respectively. The VM formation in U251 (VI) and C6 (VIII) cells was assessed using an inverted microscope after growth for another 24 h.

Glioma is an extremely invasive, well-vascularized tumor. It has been reported that vasculogenic mimicry (VM) exists in glioma [30] and *EphA2* is an important regulator for VM formation. In order to evaluate the effects of miR-26b and *EphA2* on VM formation in glioma cells, we performed VM network formation experiments in U87 MG, U251 and C6 cells. As shown in Fig. 7A, U251 and C6 glioma cells, which expressed high levels of *EphA2*, could develop VM networks when cultured in Matrigel for 24 h (Fig. 7 I and III) and the VM network showed a positive reaction with periodic acid-Shiff (PAS) (Fig. 7 II and IV). In contrast, VM network was not found in U87 MG cells which expressed low level of *EphA2* (data not shown). Our primary finding suggested that VM network formation was dependent, at least in part, on the expression of *EphA2*. Therefore, we transfected the miR-26b duplex (26b-DP) into glioma cells to determine if down-regulation of *EphA2* could affect the VM formation. As predicted, our results showed that the VM network could not be formed in either U251 or C6 cells (Fig. 7 V and VII). However, we did find that over-expression of *EphA2* fully rescued from the effects of miR-26b when a *EphA2* expression vector lacking the 3′UTR (pCMV6-XL6-EphA2) was delivered into the miR-26b over-expressed U251 and U6 cells (Fig. 7 VI and VIII). These results suggested that *EphA2* plays a key role in the VM formation, and miR-26b affects the VM formation by down-regulation of *EphA2* expression in glioma cells.

## Discussion

In the present report we detected the miR-26b expression level in human glioma samples and found that the decreased expression level of miR-26b was negatively correlated with the increased malignancy of glioma. Transfection of miR-26b duplex decreased the aggressive feature of glioma cells, suggesting that miR-26b plays a critical role in glioma development, and it may act as an anti-tumor factor in glioma cells.

Our studies indicate that *EphA2* is a novel target gene of miR-26b, and the direct interaction between miR-26b and *EphA2* mRNA is supported by several lines of evidence: (1) the 3′UTR of both human and murine *EphA2* mRNAs contain a putative binding site (the MRE) for miR-26b with significant seed match; (2) miR-26b suppresses the activity of a luciferase reporter fused with the 3′UTR of *EphA2* mRNA in an MRE dependent manner; (3) miR-26b represses the endogenous expression of human/murine *EphA2* at both the mRNA and protein level; (4) A previous study has shown that *EphA2* gene knockdown by siRNAs resulted in failure of VM formation [29]. Similar results were found in our present study, when miR-26b was over-expressed in U251 and C6 cells, the VM process was impaired, suggesting that miR-26b affects VM formation of glioma cells by down-regulating *EphA2*. This study is the first to identify a miRNA that directly regulates *EphA2*.

*EphA2* expression is frequently elevated in cancers and is associated with poor prognosis [17]–[19], [24], [31]. High levels of *EphA2* have been reported in diverse cell lines and clinical specimens, including breast, colon, prostate, non-small cell lung cancers, aggressive melanomas and glioblastoma [14], [18]. However, *EphA2* does not appear to function simply only as a biomarker [27] but also actively participated in malignant progression [17], [20], [28]. For example, ectopic expression of *EphA2* in non-transformed mammary epithelial cells is sufficient to promote a malignant phenotype as defined using in vitro and in vivo methods [19]. It has been reported that knockdown of *EphA2* in cancer cells inhibited cell malignancy and invasion [32]. In our present study, we showed that high level expression of *EphA2* was found in high grade glioma samples. Our study is consistent with other studies that high level expression of *EphA2* plays a critical role in cell malignancy. Additionally, we further confirmed that miR-26b is an important regulator of *EphA2*, and there was an interaction between miR-26b and *EphA2* in glioma cells. The inhibition induced by miR-26b in glioma cells is partly dependent on the expression of *EphA2*. This finding increases our understanding of *EphA2* function and regulation in glioma cells.

Vasculogenic mimicry (VM) was first described in highly aggressive uveal melanomas which formed vasculogenic networks with tumor cells instead of endothelial cells [33]. VM is associated with tumor blood supply and tumor metastasis. In recent years, VM has been seen in several types of malignant tumors such as breast cancer [34], liver cancer, glioma, ovarian cancer [35], melanoma [33], prostate cancer [36], colorectal cancer [37] and some other bidirectional differentiated malignant tumors [38]. It has been reported that VM also exists in glioma cells [30]; high aggressive glioma cells can form VM in three dimension culture medium [39]. *EphA2* is known to be an important regulator during VM formation [14], [29], [38], [40]. Knockdown of *EphA2* in malignant melanoma cells impaired formation of the VM network [29]. *EphA2* expression was found in both highly aggressive uveal and cutaneous melanoma cells, but not in poorly aggressive melanoma cells. Moreover, down-regulation of *EphA2* expression using a specific small interference RNA (siRNA) inhibits VM formation in aggressive melanoma cells [40]. In the present work, U251 and C6 glioma cells, which express high levels of *EphA2*, formed classical VM networks on Matrigel. In contrast, the VM networks could not be formed in U87 MG cells, which have low levels of *EphA2*. The result further suggests that *EphA2* plays a critical role in VM formation in glioma cells. We further demonstated that the function of *EphA2* is regulated by miR-26b; transfection of miR-26b duplex into U251 and C6 cells inhibited their ability to form VM networks. However, when we transfected these cells with *EphA2* expression vector without the 3′UTR regain, the VM destroyed by over-expression of miR-26b were fully restored. The formation of vasculogenic like networks has not been well understood; the processes involve several signaling molecules, including vascular endothelial (VE)-cadherin, erythropoietin-producing hepatocellular carcinoma-A2 (EphA2), phosphatidylinositol 3-kinase, focal adhesion kinase, matrix metalloproteinases and laminin 5 γ2-chain [38]. Here we found that miR-26b is a negative regulator of *EphA2* in VM formation. This finding provides insight into the function of miRNAs and *EphA2* in regulating VM processes.

In conclusion, this study demonstrates that miR-26b plays a key role in the malignancy of glioma cells by directly regulating *EphA2* expression, which affects cell proliferation, migration and invasion. VM formation was also affected by ectopic expression of miR-26b. This study helps us to better understand of the function of miR-26b and its regulation of *EphA2* in glioma cells. However, further study is needed to determine if *EphA2* activity is affected by miR-26b in other cancers with high *EphA2* expression, like breast cancer, colon cancer, and prostate cancer; and whether miR-26b-EphA2 dysregulation represents a new mechanism for cellular transformation.

## Materials and Methods

### Cell lines and tumor tissues

Human HEK-293T cells, human glioma U251 and U87 MG cells and rat glioma C6 cells were purchased from the American Type Culture Collection (ATCC, MD). All cell lines were maintained in a 37°C, 5% CO_2_ incubator in DMEM medium supplemented with 10% fetal bovine serum (Invitrogen, CA) and 1% penicillin-streptomycin (Invitrogen, CA). Cells were routinely passaged at 2 to 3 day intervals.

Tissue samples from human glioma and normal brain tissues were obtained from the Brain Institute, Qingdao Medical University Associate Hospital (Qingdao, China). Samples were collected and stored at −80°C. The histopathologic diagnoses were determined using WHO criteria and evaluated by the hospital's pathologist using both morphologic criteria and immunocytochemistry. Written consent of tissue donation for research purposes was obtained from the patients before tissue collection and the protocol was approved by the Institutional Review Board of the Affiliated Hospital of Qingdao Medical University. Twenty-five samples were used for this study with 5 samples for each group, including primary grade pilocytic astrocytomas (WHO I), grade II astrocytoma (WHO II), grade III anaplastic astrocytomas (WHO III), grade IV Glioblastoma Multiforme (WHO IV) and normal brain tissues derived from the temporal lobes and saddle area of the patients with arachnoid cyst (AC) after surgery. The clinical data and patient information are shown in Table S1. All samples were thoroughly reviewed by the neuropathologist Dr. W.C Yao in the Brain Institute, Affiliated Hospital of Qingdao Medical University (Qingdao, China).

### RNA isolation

Total RNA was extracted from the frozen tissue samples or cultured cells using the TRIzol kit (Invitrogen, CA) following the manufacturer's protocol. Briefly, tissue samples were homogenized in TRIzol reagent using an Omni-Mixer Homogenizer (Omni International, CA). The cells were collected from a culture flask into RNase free tubes, and TRIzol solution (Invitrogen, CA) was added. RNA quantity was determined by UV measurement of OD 260/280 nm using the NanoDrop 2000 instrument (Thermo Scientific, FL).

### Real-time quantitative RT-PCR

To quantitate the expression level of mature miR-26b (MIMAT0000083), the isolated RNA was reverse transcribed and amplified by a two-step quantitative RT-PCR method using the Hairpin-it TM miRNAs qPCR Quantitation Kit (Genepharma, Shanghai, China) according to the manufacturer's protocol. miR-26b specific reverse primers and the sequence-specific primers for mature miR-26b were designed according to Chen *et al.* [41] and their sequences are listed in Table 1. PCR reactions were performed using an ABI 7300 System (Bio-Rad, CA) with the following conditions: 95°C, 10 min for 1 cycle, then 95°C, 15 s, 60°C, 1 min for 40 cycles. Signals were detected at the end of each cycle. The U6 small nuclear RNA was amplified as a loading control. The primers for this U6 internal control were provided by Genepharma (Shanghai, China). The relative expression level of mature miR-26b from each sample was determined using the 2(−Delta Delta C(T) Method [42].

**Table 1:** Primer sequences.

Primer name	Type	Sequence 5′ to 3′
β-actin -F	RT PCR forward primer	GTTGCGTTACACCCTTTCTTG
β-actin -R	RT PCR reverse primer	GTCACCTTCACCGTTCCAGT
EphA2-F	RT PCR forward primer	CACTTACCGCAAGAAGGGAGA
EphA2-R	RT PCR reverse primer	ACAGCCACGCCGCCAATCA
miR-26b-RT [41]	miR-26b RT primer	GTCGTATCCAGTGCAGGGTCCGAGGTATTCGCACTGGATACGACACCTAT
miR-26b-F [41]	RT PCR forward primer	CGCCGCTTCAAGTAATTCAGGAT
miR-26b-R [41]	RT PCR reverse primer	GTGCAGGGTCCGAGGT
EphA2-MRE-F	MRE top strand	tcgagCATCGGCCAAGAATACTTGAAgc
EphA2-MRE-R	MRE bottom strand	ggccgcTTCAAGTATTCTTGGCCGATGc
EphA2-UTR-F	PCR forward primer	CCGctcgagCCTGGAGCCCCATCGGCCAAGAATA
EphA2-UTR-delF	MRE delete primer	TTCCTTTTgcggccgcCAGAGTGGCCTCCCTGCTGTGCCAT
EphA2-UTR-R	PCR reverse primer	TTCCTTTTgcggccgcAGAGCAGAAATAAGTCATTTTC
EphA2-UTR 3misF	Mutagenesis primer	GGAGCCCCATCGGCCAAGAATgtTTcAAGAAACAGAGTGG
EphA2-UTR 3misR	Mutagenesis primer	CCACTCTGTTTCTTGAAacATTcTTGGCCGATGGGGCTCC
EphA2-siRNAF [29]	EphA2 siRNA forward strand	CCAGCAGTACCGCTTCCTTGCCCTGCGGCCG
EphA2-siRNAR [29]	EphA2 siRNA forward strand	GCCGCGTCCCGTTCCTTCACCATGACGACC

Lower case text indicates restriction enzyme sites; Lower case with underline indicates mismatched mutations in miR-26b seed region.

Real-time PCR for *EphA2* was performed using the ABI 7300 System (Bio-Rad, CA) with the QuantiTect SYBR Green PCR mixture (Invitrogen, CA). *β-actin* was used as control. Primers used for detecting gene expression are listed in Table 1. Expression of *EphA2* was determined using the 2(−Delta Delta C (T) Method. Amplification conditions were as follows: 95°C, 3 min, 95°C, 30 s, 60°C, 30s, 72°C, 40s, for 40 cycles, and 72°C, 8 min for extension.

### Transfection

The two miRNA mimics used in the experiments were purchased from Dharmacon company (Dharmacon, CO), and the sequence of NC-DP microRNA, as a negative control, is based on cel-miR-67(MIMAT0000039) and it has been confirmed to have minimal sequence identity with miRNAs in human, mouse and rat. Another microRNA mimics is 26b-DP, which is a duplex of miR-26b. The antisense of two microRNAs, negative antisense of negative control microRNA, cel-miR-67 (NC-AS) and miR-26b antisense (26b-AS) were also provided by Dharmacon. Certain amount of miRNA duplexes (NC-DP and 26b-DP) and antisense oligonucleotides (NC-AS and 26b-AS) were transfected into U87 MG, U251 or C6 cells, respectively. *EphA2* expression vector (pCMV6-AC-GFP-EphA2) which contains the full length human *EphA2* gene ORF sequence without the 3′-UTR region of *EphA2* was purchased from OriGene Technologies (OriGene Technologies, MD). The pCMV6-AC-GFP-EphA2 vector was transfected into U251 or C6 cells with 20 nm/L. Specific siRNAs targeting *EphA2* (Genepharma, Shanghai, China) were transfected at 100 nm/L. Lipofection 2000 (Invitrogen, CA) was used as the transfection reagent following the manufacturer's protocol.

### Synthesis of luciferase reporter constructs

To construct the luciferase reporter vectors, the predicted miR-26b binding site (miRNA response element, MRE) on the *EphA2* 3′UTR or the whole 3′UTR of *EphA2* were inserted into the *XhoI* and *NotI* sites of a psiCheck2 vector (Promega, WI) immediate downstream of the Renilla luciferase gene. The sense and antisense sequences of the MRE were synthesized, annealed, and ligated into the psiCheck2 vector to construct a miR-26b MRE luciferase reporter, Luc+miR-26bMRE. The full length 3′ UTR of human *EphA2* was amplified from the total cDNA of U251 cells and inserted into TOPO PCR2.1 (Invitrogen, CA) and then ligated into the psiCheck2 vector to synthesize the luciferase report constructs Luc+wt *EPhA2* 3′UTR. Another construct, Luc+MRE deleted 3′UTR, in which the miR-26b MRE region of *EphA2* 3′UTR is deleted was also constructed with a similar approach. A mutation in the seed region of miR-26b MRE of the *EphA2* 3′ UTR was made using the Quick change sit-Directed mutagenesis kit (Stratagene, CA) according to the manufacturer's instructions. The construct, Luc+MRE mutated 3′UTR, was synthesized by ligating the mutation fragment into the psiCheck2 vector. The sequences of MRE and all primers are listed in Table 1.

### Luciferase reporter assay

miRNA duplexes, including the negative control duplex (NC-DP) and miR-26b duplex (26b-DP), and miRNA antisense oligonucleotide, including the negative control antisense (NC-AS) and miR-26b antisense (26b-AS) were purchased from Dharmacon and dissolved in ddH_2_O. Each luciferase reporter construct, including Luc+miR-26bMRE, Luc+wt *EPhA2* 3′UTR, Luc+MRE deleted 3′UTR and Luc+MRE mutated 3′UTR was co-transfected with 50 nm/L of miRNA duplexes or 100 nm/L miRNA antisense into HEK-293T cells in a 24-well plate using Lipofectamin-2000 (Invitrogen, CA). After incubation for 48 h, cells were harvested by centrifugation at 1000 g for 10 min and lysed using lysis buffer. Firefly and Renilla luciferase activities were determined with the Dual-Luciferase reporter system (Promega, WI) following the manufacturer's protocol.

### Western blotting analysis

U251 or C6 cells were transfected with Oligonucleotides (26b-DP or NC-DP, or 26b-AS or EphA2 siRNAs). After incubation for 72 h, the transfected cells were harvested by centrifugation at 1000 g for 10 min and lysed using ice-cold RIPA buffer (50 mM Tris–HCl, 1% NP40, 0.25% Na-deoxycholine, 150 mM NaCl, 1 mM EDTA). PMSF was added at a final concentration of 1 nm/mL. Protein concentrations were determined using the BCA protein assay kit (Thermo Scientific, FL). Whole-cell lysates (50 µg protein) were resolved by 9% SDS-PAGE and electroblotted onto nitrocellulose membranes using the Idea electrophoresis system (Idea scientific, MN). Membranes were incubated in blocking solution (1× PBS, 0.1% Tween-20, and 5% nonfat dry milk powder) for 2 h at room temperature, and then incubated for 1 h with primary antibodies at the following dilutions: Rabbit monoclonal antibodies against human *EphA2* with 1∶500 dilutions (H-20, sc-48789, Santa Cruz Biotechnology, CA) or mouse polyclonal antibody against β-actin diluted 1∶1000 (E-5, sc-47778, Santa Cruz Biotechnology, CA). After washing 10 minutes with 1× PBS and 0.1% Tween-20 for three times, membranes were incubated with horseradish peroxidase-conjugated secondary antibody (IgG goat anti-rabbit or anti-mouse; 1∶2000; Bio-Rad, CA) for 1 h at room temperature. After an additional three 10 minute washes with 0.1% Tween-20 in PBS buffer, the chemiluminescence method was employed to detect the signals using Super Signal West Dura (Thermo Scientific, FL) and protein bands were visualized by autoradiography. Quantitation of signal intensities was performed using densitometry on a Hewlett-Packard ScanJet 5370C (Hewlett-Packard, CA) with NIH image 1.62 software.

### MTT [3-(4, 5-dimethylthiazol-2-yl)-2, 5-diphenyltetrazolium bro-121 mide] assay

U251 or C6 cells (5×10^3^) were plated onto 96-well plates and allowed to adhere overnight. The cells were transfected with 26b-DP at the final concentration of 50 nm or 26b-AS at the final concentration of 100 nm using Lipofectation 2000 reagent (Invitrogen, CA). At certain time points (12, 24, 36, 48 and 72 h) after transfection, MTT dye (30 µL, 5 mg/ml, Sigma-Aldrich, MO) was added to the culture medium. After incubation at 37°C for 4 h, the MTT solution was removed and 150 µL dimethyl sulfoxide (DMSO, Sigma-Aldrich, MO) was added to dissolve the formazan crystals. Spectrometric absorbance at 490 nm was measured by Multiskan EX microplate photometer (Thermo Scientific, FL).

### Wound healing assay

Cell culture and transfection conditions were optimized to ensure a homogeneous and viable cell monolayer prior to wounding. One day before transfection, U251 (3.5×10^5^), U87 MG (3.5×10^5^) or C6 (5×10^5^) cells were seeded onto 6-well plates. Cells were then transfected with 50 nm/L 26b-DP or NC-DP using the Lipofectation 2000 transfection reagent. When the cell confluence reached about 90%, at 48 h post-transfection, an artificial homogenous wound was made onto the monolayer with a sterile plastic 200 µL micropipette tip. After wounding, cell debris was removed by washing the cells with serum-free medium. After incubation for another 24 h, the cells that migrated into the wounded area or with extended protrusion from the border of wound were photographed using an inverted microscope (40× magnifications) (CKX41, LYLMPAS, Japan).

### In vitro invasion assay

U251, U87 MG or C6 cells were plated in 6-well plates and transfected with final concentration of 50 nm 26b-DP or 100 nm 26b-AS as mentioned above. After incubation for 48 h, the cells were treated with 0.25% trypsin–EDTA (Hyclone, Japan), collected by centrifugation at 1000*g*, and re-suspended into serum-free medium. Transfected cells (5×10^4^) were reseeded into the upper chamber of transwells. 20% FBS was added to the medium in the lower chamber. After incubation for another 24 h, non-invading cells on the upper surface of the membrane were scrubbed gently with a cotton-tipped swab. The invasive cells on the lower surface of the membrane were fixed with 95% methanol and stained with 0.1% Crystal Violet (Sigma-Aldrich, MO). The stained invasive cells were photographed under an inverted light microscope (100× magnification) and quantified by manual counting in three randomly selected areas. The experiments were performed in triplicate in three independent experiments.

### Vasculogenic mimicry formation assay

Vasculogenic mimicry (VM) formation experiments were performed as previously described [33] with a little modification. Briefly, 50 µL ECM Matrigel (Sigma-Aldrich, CA) was dropped onto 18-mm glass coverlips in 6-well tissue culture plates and then incubated at 37°C for 30 min. U251 and C6 cells (5×10^5^) were transfected with 26b-DP, 26b-AS or contransfected with 26b-DP and *EphA2* expression vector (pCMV6-XL6-EphA2) a day before seeded onto the coated coverlips. After growth for 24 or 48 h on the coverlips, VM formation was assessed using an inverted microscope (CKX41, LYLMPAS, Japan). Additionally, the periodic acid-Shiff's Reaction (PAS) staining of VM formation was performed as described by Maniotis *et al.* [33]. Briefly, cells cultured on coverlips were fixed with 95% ethanol for 5 min, oxidized in 0.5% periodic acid solution for 5 min, rinsed using distilled water 3 times and placed in Schiff reagent for 15 min, and then the coverslips were immediately picked out and washed with lukewarm tap water for 5 min. The coverlips were dried at room temperature and the PAS signal was photographed using an inverted light microscope at 40× magnifications.

### Statistics

Statistical analyses were performed using the software from SPSS for Windows 13.0 (SPSS Inc., IL). All data were described as mean ± SEM. To analyze the data statistically, we performed Student's t-test for the analysis. Differences were considered significant when P<0.05.

## Supporting Information

Table S1 **Characteristics of patient tissues used in this study.** Twenty five patient tissues were used in this study and the patient tissues information were described.
(DOC)

## References

[1] Iorio MV, Visone R, Di Leva G, Donati V, Petrocca F (2007). MicroRNA signatures in human ovarian cancer.. Cancer Res.

[2] Calin GA, Croce CM (2006). MicroRNA signatures in human cancers.. Nat Rev Cancer.

[3] Olson P, Lu J, Zhang H, Shai A, Chun MG (2009). MicroRNA dynamics in the stages of tumorigenesis correlate with hallmark capabilities of cancer.. Genes Dev.

[4] Hammond SM (2007). MicroRNAs as tumor suppressors.. Nat Genet.

[5] Nicoloso MS, Spizzo R, Shimizu M, Rossi S, Calin GA (2009). MicroRNAs–the micro steering wheel of tumour metastases.. Nat Rev Cancer.

[6] Nagel S, Venturini L, Przybylski GK, Grabarczyk P, Schmidt CA (2009). Activation of miR-17-92 by NK-like homeodomain proteins suppresses apoptosis via reduction of E2F1 in T-cell acute lymphoblastic leukemia.. Leuk Lymphoma.

[7] Hyun S, Lee JH, Jin H, Nam J, Namkoong B (2009). Conserved MicroRNA miR-8/miR-200 and its target USH/FOG2 control growth by regulating PI3K.. Cell.

[8] Johnson SM, Grosshans H, Shingara J, Byrom M, Jarvis R (2005). RAS is regulated by the let-7 microRNA family.. Cell.

[9] Kim HH, Kuwano Y, Srikantan S, Lee EK, Martindale JL (2009). HuR recruits let-7/RISC to repress c-Myc expression.. Genes Dev.

[10] Cimmino A, Calin GA, Fabbri M, Iorio MV, Ferracin M (2005). miR-15 and miR-16 induce apoptosis by targeting BCL2.. Proc Natl Acad Sci U S A.

[11] Kulshreshtha R, Ferracin M, Wojcik SE, Garzon R, Alder H (2007). A microRNA signature of hypoxia.. Mol Cell Biol.

[12] Gaur A, Jewell DA, Liang Y, Ridzon D, Moore JH (2007). Characterization of microRNA expression levels and their biological correlates in human cancer cell lines.. Cancer Res.

[13] Dodelet VC, Pasquale EB (2000). Eph receptors and ephrin ligands: embryogenesis to tumorigenesis.. Oncogene.

[14] Walker-Daniels J, Hess AR, Hendrix MJ, Kinch MS (2003). Differential regulation of EphA2 in normal and malignant cells.. Am J Pathol.

[15] Salaita K, Nair PM, Petit RS, Neve RM, Das D Restriction of receptor movement alters cellular response: physical force sensing by EphA2.. Science.

[16] Ogawa K, Pasqualini R, Lindberg RA, Kain R, Freeman AL (2000). The ephrin-A1 ligand and its receptor, EphA2, are expressed during tumor neovascularization.. Oncogene.

[17] Lu C, Shahzad MM, Wang H, Landen CN, Kim SW (2008). EphA2 overexpression promotes ovarian cancer growth.. Cancer Biol Ther.

[18] Wykosky J, Debinski W (2008). The EphA2 receptor and ephrinA1 ligand in solid tumors: function and therapeutic targeting.. Mol Cancer Res.

[19] Zelinski DP, Zantek ND, Stewart JC, Irizarry AR, Kinch MS (2001). EphA2 overexpression causes tumorigenesis of mammary epithelial cells.. Cancer Res.

[20] Thaker PH, Deavers M, Celestino J, Thornton A, Fletcher MS (2004). EphA2 expression is associated with aggressive features in ovarian carcinoma.. Clin Cancer Res.

[21] Walker-Daniels J, Coffman K, Azimi M, Rhim JS, Bostwick DG (1999). Overexpression of the EphA2 tyrosine kinase in prostate cancer.. Prostate.

[22] Easty DJ, Bennett DC (2000). Protein tyrosine kinases in malignant melanoma.. Melanoma Res.

[23] Nemoto T, Ohashi K, Akashi T, Johnson JD, Hirokawa K (1997). Overexpression of protein tyrosine kinases in human esophageal cancer.. Pathobiology.

[24] Miyazaki T, Kato H, Fukuchi M, Nakajima M, Kuwano H (2003). EphA2 overexpression correlates with poor prognosis in esophageal squamous cell carcinoma.. Int J Cancer.

[25] Xu F, Zhong W, Li J, Shanshen Z, Cui J (2005). Predictive value of EphA2 and EphrinA-1 expression in oesophageal squamous cell carcinoma.. Anticancer Res.

[26] Cercone MA, Schroeder W, Schomberg S, Carpenter TC (2009). EphA2 receptor mediates increased vascular permeability in lung injury due to viral infection and hypoxia.. Am J Physiol Lung Cell Mol Physiol.

[27] Wykosky J, Gibo DM, Stanton C, Debinski W (2005). EphA2 as a novel molecular marker and target in glioblastoma multiforme.. Mol Cancer Res.

[28] Li X, Wang Y, Wang Y, Zhen H, Yang H (2007). Expression of EphA2 in human astrocytic tumors: correlation with pathologic grade, proliferation and apoptosis.. Tumour Biol.

[29] Hess AR, Seftor EA, Gardner LM, Carles-Kinch K, Schneider GB (2001). Molecular regulation of tumor cell vasculogenic mimicry by tyrosine phosphorylation: role of epithelial cell kinase (Eck/EphA2).. Cancer Res.

[30] Yue WY, Chen ZP (2005). Does vasculogenic mimicry exist in astrocytoma?. J Histochem Cytochem.

[31] Kamat AA, Coffey D, Merritt WM, Nugent E, Urbauer D (2009). EphA2 overexpression is associated with lack of hormone receptor expression and poor outcome in endometrial cancer.. Cancer.

[32] Zhou Z, Yuan X, Li Z, Tu H, Li D (2008). RNA interference targeting EphA2 inhibits proliferation, induces apoptosis, and cooperates with cytotoxic drugs in human glioma cells.. Surg Neurol.

[33] Maniotis AJ, Folberg R, Hess A, Seftor EA, Gardner LM (1999). Vascular channel formation by human melanoma cells in vivo and in vitro: vasculogenic mimicry.. Am J Pathol.

[34] Shirakawa K, Kobayashi H, Heike Y, Kawamoto S, Brechbiel MW (2002). Hemodynamics in vasculogenic mimicry and angiogenesis of inflammatory breast cancer xenograft.. Cancer Res.

[35] Wang JY, Sun T, Zhao XL, Zhang SW, Zhang DF (2008). Functional significance of VEGF-a in human ovarian carcinoma: role in vasculogenic mimicry.. Cancer Biol Ther.

[36] Sharma N, Seftor RE, Seftor EA, Gruman LM, Heidger PM (2002). Prostatic tumor cell plasticity involves cooperative interactions of distinct phenotypic subpopulations: role in vasculogenic mimicry.. Prostate.

[37] Baeten CI, Hillen F, Pauwels P, de Bruine AP, Baeten CG (2009). Prognostic role of vasculogenic mimicry in colorectal cancer.. Dis Colon Rectum.

[38] Zhang S, Zhang D, Sun B (2007). Vasculogenic mimicry: current status and future prospects.. Cancer Lett.

[39] Le Mercier M, Fortin S, Mathieu V, Roland I, Spiegl-Kreinecker S (2009). Galectin 1 proangiogenic and promigratory effects in the Hs683 oligodendroglioma model are partly mediated through the control of BEX2 expression.. Neoplasia.

[40] Hess AR, Margaryan NV, Seftor EA, Hendrix MJ (2007). Deciphering the signaling events that promote melanoma tumor cell vasculogenic mimicry and their link to embryonic vasculogenesis: role of the Eph receptors.. Dev Dyn.

[41] Chen C, Ridzon DA, Broomer AJ, Zhou Z, Lee DH (2005). Real-time quantification of microRNAs by stem-loop RT-PCR.. Nucleic Acids Res.

[42] Livak KJ, Schmittgen TD (2001). Analysis of relative gene expression data using real-time quantitative PCR and the 2(−Delta Delta C(T)) Method.. Methods.

