# Supplementary material for: Role of MicroRNA-26b in Glioma Development and Its Mediated Regulation on EphA2
Source: PLoS One. 2011 Jan 14;6(1):e16264. doi: 10.1371/journal.pone.0016264 (PMC3021542; doi:10.1371/journal.pone.0016264)
Supplement: Table S1 — Characteristics of patient tissues used in this study. Twenty five patient tissues were used in this study and the patient tissues information were described. (DOC) [file pone.0016264.s001.doc]

| Sample designation | Sample ID | Age | Sex | Histology type | WHO Grade |
| --- | --- | --- | --- | --- | --- |
| AC-683649 | AC-1 | 20 | M | Arachnoid Cyst (AC) | 0 |
| AC-634574 | AC-2 | 45 | F | Arachnoid Cyst (AC) | 0 |
| AC-668854 | AC-3 | 25 | M | Arachnoid Cyst (AC) | 0 |
| AC-638363 | AC-4 | 29 | F | Arachnoid Cyst (AC) | 0 |
| AC-649532 | AC-5 | 42 | M | Arachnoid Cyst (AC) | 0 |
| AP-699954 | AP-1 | 9 | M | Astrocytomas Pilocytic (AP) | Ⅰ |
| AP-697559 | AP-2 | 13 | M | Astrocytomas Pilocytic (AP) | Ⅰ |
| AP-672466 | AP-3 | 27 | F | Astrocytomas Pilocytic (AP) | Ⅰ |
| AP-647542 | AP-4 | 21 | M | Astrocytomas Pilocytic (AP) | Ⅰ |
| AP-682035 | AP-5 | 32 | F | Astrocytomas Pilocytic (AP) | Ⅰ |
| AD-699809 | AD-1 | 72 | F | Astrocytomas,Diffuse (AD) | Ⅱ |
| AD-699583 | AD-2 | 34 | M | Astrocytomas,Diffuse (AD) | Ⅱ |
| AD-450636 | AD-3 | 43 | F | Astrocytomas,Diffuse (AD) | Ⅱ |
| AD-696783 | AD-4 | 21 | F | Astrocytomas,Diffuse (AD) | Ⅱ |
| AD-697868 | AD-5 | 21 | M | Astrocytomas,Diffuse (AD) | Ⅱ |
| AA-697099 | AA-1 | 33 | M | Astrocytoma, Anaplastic (AA) | Ⅲ |
| AA-695016 | AA-2 | 54 | M | Astrocytoma, Anaplastic (AA) | Ⅲ |
| AA-695707 | AA-3 | 46 | M | Astrocytoma, Anaplastic (AA) | Ⅲ |
| AA-699114 | AA-4 | 34 | F | Astrocytoma, Anaplastic (AA) | Ⅲ |
| AA-698882 | AA-5 | 46 | F | Astrocytoma, Anaplastic (AA) | Ⅲ |
| GMB-682617 | GBM-1 | 70 | M | Glioblastoma Multiforme (GBM) | Ⅳ |
| GMB-683427 | GBM-2 | 44 | F | Glioblastoma Multiforme (GBM) | Ⅳ |
| GMB-683915 | GBM-3 | 66 | F | Glioblastoma Multiforme (GBM) | Ⅳ |
| GMB-692495 | GBM-4 | 53 | M | Glioblastoma Multiforme (GBM) | Ⅳ |
| GMB-697095 | GBM-5 | 46 | M | Glioblastoma Multiforme (GBM) | Ⅳ |

Suplement Table 1. Characteristics of patient tissues used in this study
